# Isolation and Characterization of a Mn(II)-Oxidizing *Bacillus* Strain from the Demosponge *Suberites domuncula*

**DOI:** 10.3390/md9010001

**Published:** 2010-12-23

**Authors:** Xiaohong Wang, Matthias Wiens, Mugdha Divekar, Vladislav A. Grebenjuk, Heinz C. Schröder, Renato Batel, Werner E. G. Müller

**Affiliations:** 1 National Research Center for Geoanalysis, 26 Baiwanzhuang Dajie, CHN-100037 Beijing, China; 2 Institute for Physiological Chemistry, Dept. for Applied Molecular Biology, Johannes Gutenberg-University Medical Center, Duesbergweg 6, D-55099 Mainz, Germany; E-Mails: wiens@uni-mainz.de (M.W.); mugdhadivekar03@yahoo.co.in (M.D.); grebenyu@mail.uni-mainz.de (V.A.G.); hschroed@mail.uni-mainz.de (H.C.S.); 3 Center for Marine Research, “Ruder Boskovic” Institute, HR-52210 Rovinj, Croatia; E-Mail: batel@cim.irb.hr (R.B.)

**Keywords:** manganese precipitating bacteria, BAC-SubDo-03, *Suberites domuncula*, multicopper oxidase, Mn storage

## Abstract

In this study we demonstrate that the demosponge *Suberites domuncula* harbors a Mn(II)-oxidizing bacterium, a *Bacillus* strain, termed BAC-SubDo-03. Our studies showed that Mn(II) stimulates bacterial growth and induces sporulation. Moreover, we show that these bacteria immobilize manganese on their cell surface. Comparison of the 16S rDNA sequence allowed the grouping of BAC-SubDo-03 to the Mn-precipitating bacteria. Analysis of the spore cell wall revealed that it contains an Mn(II)-oxidizing enzyme. Co-incubation studies of BAC-SubDo-03 with 100 μM MnCl_2_ and >1 μM of CuCl_2_ showed an increase in their Mn(II)-oxidizing capacity. In order to prove that a multicopper oxidase-like enzyme(s) (MCO) exists in the cell wall of the *S. domuncula-*associated BAC-SubDo-03 *Bacillus* strain, the gene encoding this enzyme was cloned (*mnxG-SubDo-03*). Sequence alignment of the deduced MCO protein (MnxG-SubDo-03) revealed that the sponge bacterium clusters together with known Mn(II)-oxidizing bacteria. The expression of the *mnxG-SubDo-03* gene is under strong control of extracellular Mn(II). Based on these findings, we assume that BAC-SubDo-03 might serve as a Mn reserve in the sponge providing the animal with the capacity to detoxify Mn in the environment. Applying the *in vitro* primmorph cell culture system we could demonstrate that sponge cells, that were co-incubated with BAC-SubDo-03 in the presence of Mn(II), show an increased proliferation potential.

## 1. Introduction

It is amazing that on the deep-sea floor in depths of around 4,000–6,000 m manganese (Mn) can be deposited in polymetallic nodules despite very low concentrations in the surrounding environment [1] of only approximately 0.2 nmol/kg. It has been reported that in this biotope, bacteria concentrate this element to a level of about 20 g/100 g, e.g., in polymetallic nodules or crusts [2]. Very recently we were able to demonstrate, by application of high-resolution scanning electron microscopy, that inside the mineralized deposits of the polymetallic nodules, imprints from bacteria can be traced, and not on their surfaces [3,4]. This finding implies that the bacteria must have been buried alive during Mn deposition in the growing nodules. Further support for a biogenic factor in nodule formation, more specifically for the involvement of bacteria in the initial phase of nodule formation, came from studies which showed that the bacteria in the polymetallic nodules are decorated with S-layer structures [5,6]. It has been shown in thorough studies that a variety of phylogenetic distantly related, free-living bacteria, are able to oxidize and metabolize Mn(II) through their multicopper oxidase-like enzyme(s) (MCO) to Mn(IV) [7]. The MCOs are able to reduce the activation energy required for the oxidation of Mn(II) to Mn(III) and Mn(IV), and allow the release of an appreciable amount of free energy (ΔG −50 kJ/mol) during the oxidation process. Most data on these enzymes have been obtained from Mn(II)-oxidizing bacteria such as *Pseudomonas putida* [8], *Leptothrix discophora* [9], *Bacillus* sp. strain [10–14], and *Pedomicrobium* sp [15]. MCOs use multiple Cu atoms as cofactors that are required for the coupled oxidation of a series of substrates [7]. The MCOs have been implicated in the oxidation of organic metal-containing compounds and of metal ions, e.g., Fe(II) and Mn(II) [16]. In extensive studies the group of Tebo [11–13] succeeded in providing direct evidence for the role of the MCO during Mn-deposition on bacteria, both on an enzymic and at a molecular level. It has been shown that besides the MCOs heme peroxidase oxidizes in some bacteria manganese [17].

In the present study, we identified for the first time a *Bacillus* strain of Mn(II)-oxidizing bacteria in the demosponge *Suberites domuncula*. This sponge species is especially suitable for the identification of potential symbiotic microorganisms since it can be kept under controlled laboratory conditions for over five years [18]. The data revealed that these Mn(II)-oxidizing bacteria are closely related to the Mn(II)-oxidizing *Bacillus* strains isolated from the Guaymas Basin, a deep-sea hydrothermal vent environment in the Gulf of California [11]. On the basis of recently published data [12,13], the *mnxG* gene coding for the MCO has also been identified in the newly discovered *S. domuncula*-associated bacteria. The expression of this gene was found to depend on the presence of Mn in the culture medium. We propose that the sponge-associated *Bacillus* strain enables storage of Mn in *S. domuncula*. It is assumed that the bacterial MCO is the central enzyme that controls the homeostasis of Mn in the sponge. At lower environmental Mn concentrations, MCO triggers accumulation of Mn on the surfaces of the bacteria, and hence in the body of the sponge to physiological levels, while at higher ambient Mn concentrations in the water; the enzyme protects the sponge against toxic effects of Mn. In order to support this new view on the role of Mn-oxidizing bacteria in animals, we performed co-incubation studies of sponge cells with the *S. domuncula*-associated Mn(II)-oxidizing bacteria. Furthermore, to assess their (potential) function on cell metabolism we co-incubated primmorphs [19], three dimensional (3D) sponge cell aggregates, with those Mn(II)-oxidizing bacteria. It was found that sizes and gene expression levels of the primmorphs increased if such cultures were incubated in the presence of Mn(II).

## 2. Results

### 2.1. Isolation of Mn(II)-Oxidizing Bacillus Strain BAC-SubDo-03

**Figure 1:**
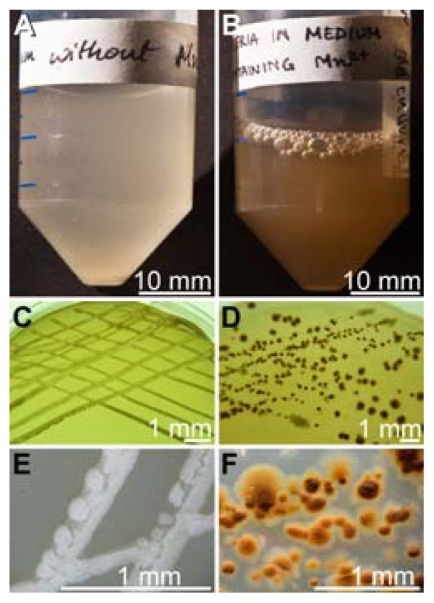
Growth of the Mn(II)-oxidizing bacterium strain BAC-SubDo-03 isolated from *S. domuncula*. Extracts from the sponge were prepared and plated on Mn agar. Colonies of Mn(II)-oxidizing microorganisms were picked and subsequently cultivated (7 d) in liquid K-medium. This medium either lacked Mn (**A**), or was supplemented with Mn (100 μM MnCl_2_) (**B**). (**C**) Cultivation (for 72 hrs) of strain BAC-SubDo-03 on agar, lacking Mn or (**D**) containing 100 μM MnCO_3_. (**E**) At higher magnification it is apparent that on agar medium without Mn the colonies were whitish, whereas on agar supplemented with Mn the colonies exhibited a brownish area around the colonies indicating Mn deposition (**F**).

Mn(II)-oxidizing bacteria were isolated from tissue of *S. domuncula* during a selection procedure on Mn agar plates in modified K-medium, containing 100 μM MnCl_2_, as described under “Experimental Section”. The isolated strain BAC-SubDo-3 was found to oxidize MnCl_2_ (present in the K-medium) or MnCO_3_ (in the agar) as deduced from the color change. In the absence of Mn in the culture system the medium (Figure 1A), as well as the colonies on the agar (Figure 1C), remained whitish. In contrast, in Mn-containing liquid cultures (Figure 1B) or on agar (Figure 1D) a shift from whitish to brownish was seen. The morphology of the bacterial streaks on agar changed during the process of Mn precipitation. While in the absence of Mn in the agar, the streaks were more or less continuous (Figure 1E) the colonies on Mn-containing agar formed islands of sizes of around 50 μm^2^ surrounded by Mn deposits (Figure 1F).

**Figure 2:**
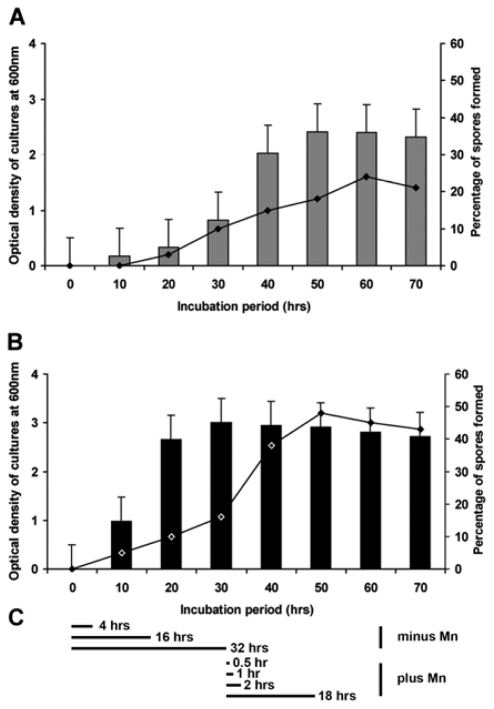
Growth kinetics and spore formation of strain BAC-SubDo-03 in K-medium (pH 6.8) in absence (**A**) or presence (**B**) of 100 μM MnCl_2_. Bacterial growth was measured spectrophotometrically as an increase of optical density at 600 nm [bars] (mean values and standard deviations [SD] from five independent experiments are given). The percentage of spores (sporangia, free spores) was determined microscopically after staining of the samples with malachite green/safranin; these values are given as dots connected by a line. (**C**) Scheme representing the time points at which bacteria were harvested to determine the expression level of the *mnxG* gene by polymerase chain reaction (PCR) in the *Bacillus* strain SubDo-03 in absence (minus Mn) or presence of 100 μM MnCl_2_ (plus Mn). The data of the respective RT-PCR reactions are given in Figure 9.

The growth rate of strain BAC-SubDo-3 depended on the presence of MnCl_2_ in the medium. In the absence of any Mn a longer lag phase of 20 to 30 hours was observed [the optical density of the 30 hours-old cultures reached a value of 0.8 units] followed by a short (from 30 to 40 hours) increase in cell density, prior to the stationary phase which was reached after 40 hours (Figure 2A). Addition of 100 μM MnCl_2_ shortened the lag phase to 6 to 10 hours (the optical density after 30 hours: 2.95 units); the stationary phase was reached already after 16 to 20 hours (Figure 2B).

### 2.2. Phylogenetic Grouping of BAC-SubDo-03

**Figure 3:**
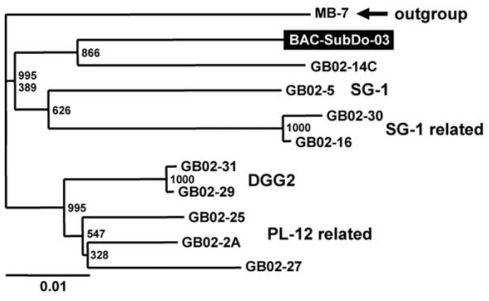
Rooted neighbor-joining phylogenetic tree based on the 16S rDNA sequences of published Mn(II)-oxidizing Bacillus species and related microorganisms [11], and the *S. domuncula* Mn(II)-oxidizing microorganism (strain BAC-SubDo-03). The following published sequences were selected. The Mn(II)-oxidizing bacteria GB02-14C (DQ079004); the sequences from GB02-25 (DQ079010), GB02-2A (DQ079008) and GB02-27 (DQ078997) belonging to the PL-12 related cluster; from the SG-1 cluster (collected at hydrothermal vents; [20]) the *Bacillus* species GB02-5 (DQ078996), GB02-30 (DQ079007) and GB02-16 (DQ079006) and finally, from the hot spring clones DGG2, the clones GB02-31 (DQ079000) and GB02-29 (DQ079002). The sequence, from Bacillus species MB-7 (AF326364), falling within the “halo” cluster [11], was used as outgroup. Bootstrap values based on 1,000 replicates, is indicated at the branch points.

The Mn(II)-oxidizing *Bacillus* strain BAC-SubDo-03 was phylogenetically grouped by analyzing its 16S rDNA (*16S-BAC-SubDo-03*) by comparison with the data bases. Highest sequence similarity was found with the Mn(II)-oxidizing spores isolated from the Guaymas Basin [11], and most closely related to strain GB02-14C (Figure 3).

### 2.3. Formation of Spores

**Figure 4:**
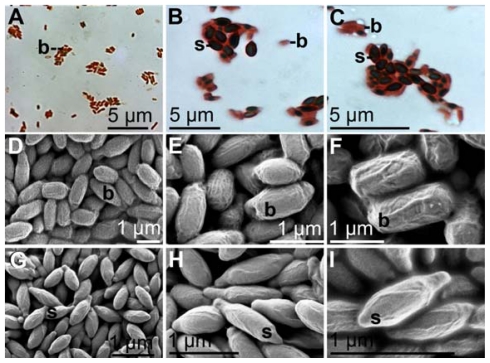
Spore formation of strain BAC-SubDo-03. The bacteria (**b**) were cultivated in Mn-containing K-medium. (**A** to **C**). Cultures were incubatred for 24 hours in Mn-containing medium (100 μM MnCl_2_) at 28 °C, pH 6.8, to induce spore formation. The cells were stained with malachite green/safranin. The spores (**s**) appear in dark, while the bacteria (**b**) light up in red. (**D** to **F**) SEM image of a culture grown in K medium without additional Mn(II) after 8 hours of incubation at 28 °C, pH 6.8. (**G** to **I**) Samples, when grown in presence of added Mn(II) for 18 hours showed the microorganisms with elongated spindle shaped structures, spores/endospores (**s**). SEM images.

In previous studies it was indicated that primarily spores have the capacity to oxidize Mn(II) to Mn(IV) [21,22]. Therefore, we determined in the BAC-SubDo-03 cultures, the fraction of spores throughout the growth cycle of the bacteria. The spores were identified by staining with malachite green followed by counterstaining with safranin. During these reactions the spores stained dark-red/green (Figure 4A to C).

In the absence of MnCl_2_, first spores could be detected after 20 hours of incubation in K-medium, and the fraction increased steadily until the maximum with 20% was reached after 60 hours (Figure 2A). In contrast, in the presence of 100 μM of MnCl_2_ spore-formation started already after 10 hours and reached the maximum of 40% already after 40 hours of incubation (Figure 2B).

SEM images were taken from BAC-SubDo-03 cultures grown for 10 hours in the absence or presence of MnCl_2_. In the absence of MnCl_2_ all bacteria have a barrel-like morphology (Figure 4D to F). In contrast, cultures that were incubated for 10 hours in the presence of 100 μM of MnCl_2_ contained bacteria with a different morphology. Those microorganisms had distinct elongated projections that are labeled here as endospores/spores (Figure 4G to I). In parallel, the cells were stained with malachite green/safranin (Figure 4A to C).

### 2.4. Detection of Mn-Oxidizing Activity: In-Gel Determination

**Figure 5:**
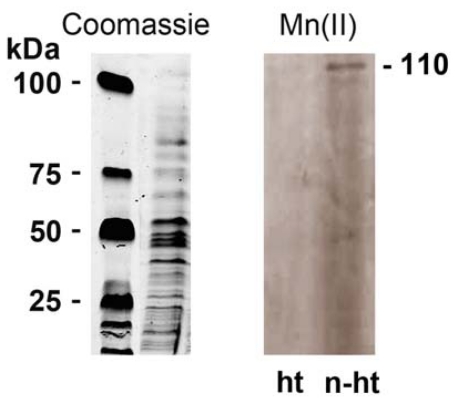
*In situ* identification of Mn-oxidizing activity in the outermost spore layer(s). Samples (10 μg of protein) were subjected to SDS-PAGE as described under “Experimental Section”. **Left panel**: Staining of total protein by Coomassie brilliant blue. Samples from spores developed in cultivation medium supplemented with 100 μM of MnCl_2_ were analyzed. **Right panel**: The same extracts were analyzed for Mn-oxidizing activity. After size-separation the gel was treated in HEPES buffer containing 10% glycerol and 0.5% Triton X-100 for 30 min to remove NaDodSO_4_ prior to incubation with 100 μM Mn(II) for 2 hrs to stain for Mn-oxidizing activity using the *in situ* (in-gel) oxidation activity assay. The samples applied onto the gel were either heat-treated (96 °C, 5 min) or left unheated (n-ht). A 110 kDa band developed from the unheated sample (right lane), whereas no 110 kDa band became visible from the heat-treated sample.

The Mn-oxidizing activity was determined in cell wall extracts from BAC-SubDo-03 endospores after cultivation of the bacteria in K-medium in the presence of 100 μM MnCl_2_ for 24 hours. The outermost layers (cell walls) from the spores were obtained by physical treatment, as described in “Experimental Section”, and the proteins were analyzed by SDS-PAGE. The gels were either directly stained with Coomassie brilliant blue (Figure 5; left panel) or incubated in the presence of 100 μM MnCl_2_ for the identification of the Mn-oxidizing activity *in situ* (Figure 5; right panel). For this *in situ* determination assay, the samples were either left unheated (n-ht) or were heated at 96°C for 5 minutes (ht). Evidently a 110-kDa protein band developed only from the unheated sample (Figure 5, right panel [n-ht], whereas no band became visible from the heated sample (Figure 5, right panel [ht]). In an additional control, it was shown that no 110-kDA band appeared when the gels were incubated in the presence of 100 μM MnCl_2_ plus 10 mM Na-azide (data not shown). This finding is consistent with experimental data that indicated the involvement of a metalloprotein (multicopper oxidase) in Mn-oxidation [16].

### 2.5. Level of Mn-Oxidizing Activity Depending on the Presence of Mn in the Medium

**Figure 6:**
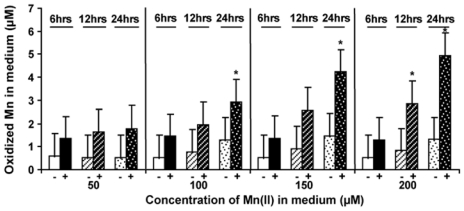
Capacity of the sponge associated BAC-SubDo-03 bacteria to oxidize Mn(II) in the medium. The bacterial cultures (+ bacteria; darker shaded columns) were incubated in K medium with increasing concentrations of MnCl_2_ (50 μM to 200 μM) for 6, 12 or 24 hrs, as indicated. In parallel, medium was supplemented with MnCl_2_ for the same period of time. As a control, uninoculated medium (-bacteria; brighter shaded bars) with added MnCl_2_ was run in parallel with the inoculated medium. Five independent experiments were performed and the mean values (means and SD) are shown; significance: *P < 0.001. Aliquots (2 mL) from the cultures were assayed for the concentration of the oxidized manganese in the medium. The amount of oxidized Mn(II) was determined by the colorimetric dye leucoberberlin blue which reacts with Mn(III) and Mn(IV). The respective optical densities were converted to μM of Mn(IV) by using K-permanganate as reference material.

Cultures of BAC-SubDo-03 grown in K-medium supplemented with different concentrations of MnCl_2_ for periods of six to 24 hours were analyzed for their capacity to oxidize Mn(II) by the leucoberbelin blue reagent assay. The data revealed that cultures grown in the absence of MnCl_2_ showed a low Mn(II)-oxidizing activity that ranged between 0.5 to 1.0 μM of oxidized Mn(II) (Figure 6). However, when the bacteria were grown in the presence of MnCl_2_ a time- and concentration-dependent increase in the Mn(IV)-product formation was measured. While the increase of the Mn(II)-oxidizing activity at a concentration of 50 μM of MnCl_2_ was not significant during the 6 to 24 hours incubation period a significant increase in oxidation was seen in cultures that were incubated with 100 μM MnCl_2_ for 24 hours. Higher concentrations of MnCl_2_ (150 μM and 200 μM MnCl_2_) resulted in a further increase of the activity. At the highest concentration tested (200 μM MnCl_2_), the cells formed 2.8 μM oxidized Mn after incubation for only 12 hours (Figure 6). This value even increased when the incubation of the bacteria was extended to 24 hours (5 μM).

Based on this finding, we conclude that Mn(II) in the culture medium induced the expression of the MCO in a time- and concentration-dependent manner.

### 2.6. Binding/Oxidation of Manganese by the BAC-SubDo-03 Strain

**Figure 7:**
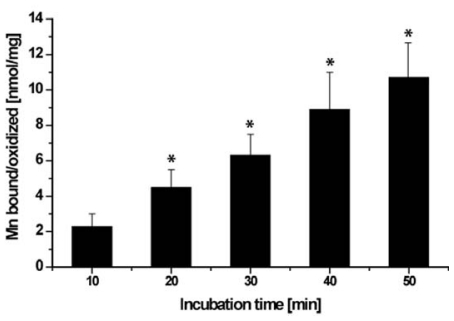
Binding of manganese to BAC-SubDo-03 bacteria. The microorganisms were incubated in K medium, supplemented with radiolabeled manganese, as described under “Experimental Section”. Then aliquots were taken and the activity was determined. The total amount of manganese immobilized by the bacteria is given in nmol/mg of bacterial protein. The mean values, together with SD, from five independent experiments are given; significance: *P < 0.001.

In view of earlier studies [23] we performed experiments to also determine the amount of manganese bound to or oxidized by the microorganisms BAC-SubDo-03. Therefore these bacteria were cultivated in medium containing radiolabeled manganese for up to 50 minutes (Figure 7). Then aliquots were taken from the cultures and the radioactivity was determined. The data revealed that the total amount of manganese immobilized on the bacteria increased almost linearly during the 50 minute incubation. After 50 minutes 10.7 ± 1.9 nmoles of Mn were associated with bacteria based on mg of protein.

### 2.7. Effect of Copper on Manganese Oxidation

**Figure 8:**
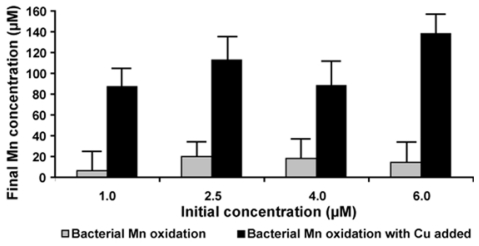
Effect of copper ions on the enzyme activity of BAC-SubDo-03 cultures. Cultures were allowed to oxidize Mn(II) in the absence (grey bars) or presence (black bars) of copper ions (as CuCl_2_) up to a concentration of 5 μM added to the medium for 12 hrs at 28 °C. The Mn concentration was determined colorimetrically. The mean values and SDs from five experiments are presented. The increase of the activity caused by Cu ions is significant at all 4 concentrations of CuCl_2_ used; *P < 0.001.

In order to test whether copper ions are able to stimulate Mn-oxidation activity of BAC-SubDo-03, the bacterial cells were incubated in the presence of 200 μM of MnCl_2_ (like in the experiment summarized in Figure 6) together with increasing concentrations of Cu ions (1.0 to 6.0 μM CuCl_2_). As expected, the Mn-oxidizing activity in parallel cultures incubated in the presence of 200 μM of MnCl_2_ varied between 4 to 10 μM during the 24 hours incubation (Figure 8). However, if Cu ions (1.0 μM) were added, the activity increased drastically to 90 μM during the 24 hours of incubation. This level rose to 138 μM when the concentration of Cu ion was increased to 6 μM (Figure 8). This result supports further the notion that a MCO exists on the surface of the bacterial endospores.

### 2.8. *mnxG* Gene in BAC-SubDo-3

**Figure 9:**
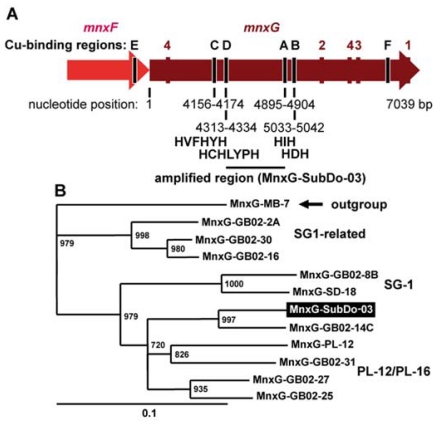
Sequence relationship of the *mnxG* gene (termed *mnxG-SubDo-03*), identified in the *Bacillus*-related strain, isolated from *S. domuncula*. (**A**) Schematic representation of the *mnx* region with the central gene MnxG-SubDo-03 attributed to the MCO, taken from Dick *et al.* [11]. The locations of the putative Cu-binding regions within MnxG-SubDo-03, the regions E to F, have been indicated. The primers to identify the MCO from the sponge-associated bacteria (BAC-SubDo-03) span the regions C to B; the respective locations are indicated using the numbering of the gene isolated from the bacterial strain SG-1 [12]. The consensus Cu-binding sequence motifs (C/D and A/B) found in all MCOs are given. (**B**) The polypeptide, MnxG-SubDo-03, deduced from the partial nucleotide sequence (*mnxG-SubDo-03*) was compared with the MnxG proteins from the following *Bacillus* strains [11–13]: strain PL-12 (MnxG-PL-12; ABP68890.1), strain GB02-31 (MnxG-GB02-31; AAZ31744.1), strain GB02-30 (MnxG-GB02-30; AAZ31743.1), strain GB02-27 (MnxG-GB02-27; AAZ31742.1), strain GB02-25 (MnxG-GB02-25; AAZ31741.1), strain GB02-16 (MnxG-GB02-16; AAZ31739.1), strain GB02-14C (MnxG-GB02-14C; AAZ31738.1), strain GB02-8B (MnxG-GB02-8B; AAZ31736.1), strain SD-18 (MnxG-SD-18; AAL30449.1), strain GB02-8B (MnxG-GB02-8B; AAZ31736), strain GB02-18 (MnxG-GB02-18; AAL30449.1) and strain GB02-2A (MnxG-GB02-2A; AAZ31735.1). These sequences were aligned, the tree computed and rooted with the sequence from *Bacillus* strain MB-7 (ABP68890) [see Figure 3]. The bootstrap values base on 1,000 replicates and are indicated at the branch points. The grouping of the sequences was performed as outlined by Dick *et al.* [11] and in Figure 3.

The published sequences from the multicopper oxidases were used to design degenerate primers [11,12], which then were applied successfully to identify the corresponding oxidase of the BAC-SubDo-03 strain. The sequence was termed *mnxG-SubDo-03*. The deduced sequence fragment was 235 amino acids in length and spanned the central part of the putative MnxG Cu-binding regions D and A/B [13]; Figure 9A. The consensus amino acids involved in the binding of the MCO to Cu are highlighted under the respective domains C/D and A/B (Figure 9A). To elucidate the relationship with other enzymes the MnxG-SubDo-03 polypeptide deduced from the *mnxG-SubDo-03* sequence was compared with other oxidases involved in Mn-oxidation [11–13]. This analysis revealed that the sponge/microorganism-related oxidase (MnxG-SubDo-03) shared the highest sequence relationship with the oxidase from the *Bacillus* strain GB02-14C (Figure 9B). These two sequences formed one branch together with the PL-12/PL-16 cluster.

The MCO-specific primers were also used to check if, in the seawater in which the *S. domuncula* specimens live, BAC-SubDo-03 or related strains could be identified. Seawater was taken, centrifuged, and the sediment was suspended in the specific PCR reaction mixture supplemented with the specific primers for the *MnxG* gene. However, no bacteria containing the *MCO* gene were found in any one of the six separate experiments. In continuation—and in order to get a hint as to whether those bacteria were absent in the surrounding seawater—we applied the cetyltrimethylammonium bromide (CTAB) extraction procedure, described originally by us [24]. Again no signals were obtained after applying the PCR technique to screen for the existence of the *MCO* gene (data not shown).

### 2.9. Induction of *mnxG* Gene after Incubation with MnCl_2_

**Figure 10:**
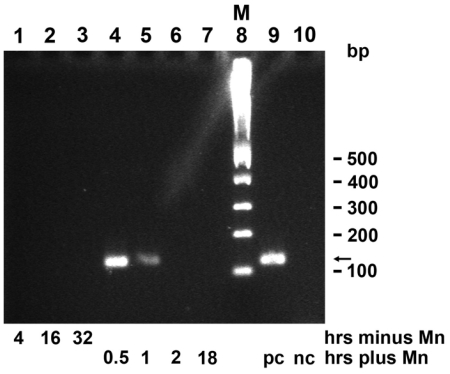
Expression of *mnxG* gene in BAC-SubDo-03, grown, in absence of Mn (minus), or presence of Mn (plus). As described under “Experimental Section”, and schematically outlined in Figure 2C, the cultures were incubated for 4 hrs, 16 hrs, and 32 hrs (**lanes 1 to 3**) in the absence of Mn; or, the microorganisms were cultivated in the absence of Mn and then exposed for 0.5 hr, 1 hr, 2 hr or 18 hrs to 100 μm MnCl_2_ (**lanes 4 to 7**). The same quantity of bacteria was taken after the indicated period of incubation. Then, RNA was extracted and the level of transcripts was semi-quantitatively determined by RT-PCR. The molecular size markers (M) are shown in **lane 8**. In parallel, amplifications by RT-PCR in an assay supplemented with the *mnxG-SubDo-03* containing plasmid (positive control [pc]), or with an empty pGEM-T vector (negative control [nc]). This PCR gel analysis is representative for five separate analyses performed.

In a semi-quantitative approach, the expression level of the *mnxG* gene in BAC-SubDo-03 (*mnxG-SubDo-03*) was determined by RT-PCR in the absence or presence of Mn. The schedule of Mn treatment is outlined in Figure 2C. In the absence of Mn in the culture medium, no signals for *mnxG-SubDo-03* transcripts were detected during an incubation period of 4 to 32 hours (Figure 10; lanes 1 to 3). However, when cultures that had been grown in the absence of Mn for 32 hours were then exposed to 100 μM MnCl_2_, a strong 119 bp long band reflecting the expected size of the amplified stretch of the *mnxG-SubDo-03* transcripts could be identified after ethidium bromide staining (Figure 10; lane 4). The level of transcripts could still be recognized in BAC-SubDo-03 after a MnCl_2_ pulse of 1 hour (lane 5), but not anymore after 18 hours of exposure (lane 7). In parallel, amplifications the assays contained either the *mnxG-SubDo-03* containing plasmid (positive control [pc]) or the empty plasmid (negative control [nc]); lanes 9 and 10. Controls were performed with samples not subjected to RT reaction, but no amplification signals were obtained (not shown).

### 2.10. Co-Incubation of *S. domuncula* Primmorphs with BAC-SubDo-03

**Figure 11:**
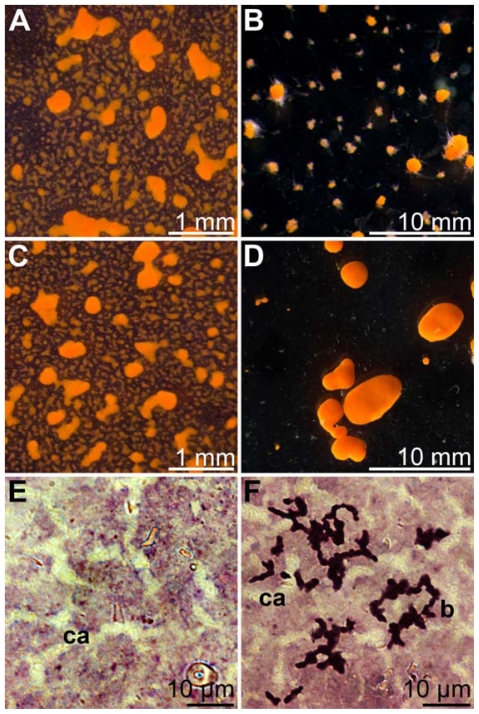
Co-incubation of *S. domuncula* primmorphs composed of proliferating cells in 3D direction with Mn-oxidizing bacteria BAC-SubDo-03. Dissociated cells from *S. domuncula* were allowed to re-associate *in vitro* under formation of primmorphs displaying a smooth surface. The dissociated cells were transferred into culture medium which remained free of Mn, or which had been supplemented with 50 μM MnCl_2_. After one day in culture, small cell clumps were formed from cultures devoid of Mn (**A**), and cultures grown in the presence of MnCl_2_ (**C**). Then, aliquots of primmorphs were further cultivated in Mn-free, or Mn-supplemented medium, and remained either non-infected with BAC-SubDo-03, or were infected with these bacteria. After additional cultivation for three days, primmorphs were formed that remained relatively small if kept in Mn-free medium and after inoculation with BAC-SubDo-03 (**B**). However, if the primmorphs that had been co-incubated with BAC-SubDo-03 were cultivated in Mn-containing medium, they grew to larger-size primmorphs (**D**). Histological analyses of primmorphs for the presence of bacteria. Sections were performed through non-inoculated primmorphs (**E**), or (**F**) through 3D-cell aggregates that had been inoculated with BAC-SubDo-03 and then cultured for 3 days. The latter sections showed band-like colonies of bacteria (b) beside the canal-like structures (ca). The sections (E and F) were stained with hematoxylin/eosin

In order to analyze the (potential) effect of the Mn-oxidizing bacteria BAC-SubDo-03 on sponge cell metabolism, the sponge 3D-cell cultures were inoculated with the microorganisms, as described under “Experimental Section”. After dissociation of the sponge tissue, the single cell suspension was allowed to re-aggregate to clumps of approximately 400–600 μm in diameter for one day in the absence or presence of MnCl_2_ (Figure 11). The sizes of the cell clumps in both assays, in the absence or the presence of 50 μM MnCl_2_, did not differ (6 parallel experiments have been performed); Figure 11A and C. Then the 3D-cell culture samples remained non-inoculated or were inoculated with BAC-SubDo-03 bacteria, and were incubated in the absence or presence of 50 μM MnCl_2_ for an additional three days. During that period the 3D-cell clumps formed primmorphs with smooth surfaces. While primmorphs not incubated with BAC-SubDo-03 grown in the absence or presence of MnCl_2_, or primmorphs inoculated with BAC-SubDo-03 and growing in MnCl_2_-free medium, reached only diameters between 2 and 3 mm (Figure 11B); those primmorphs which had been inoculated with BAC-SubDo-03 and cultured in the presence of 50 μM MnCl_2_ reached sizes of 5–6 mm (Figure 11D).

Histological analyses ensured that the bacteria had colonized the primmorphs. The primmorph slices were stained with hematoxylin/eosin in order to visualize and contrast the canal-like structures [25]. With this procedure no bacteria could be identified in primmorphs which had not been inoculated with BAC-SubDo-03 (Figure 11E). This was in contrast to primmorphs which had been co-incubated with BAC-SubDo-03; slices from those 3D-cell aggregates comprised deeply stained band-like colonies (Figure 11F). It should be mentioned here that the sponge *S. domuncula* does not abundantly contain large communities of bacteria in its body [26]. In contrast to other sponge-associated bacteria that are in *S. domuncula* “encapsulated” in special sponge cells, bacteriocytes [26], the BAC-SubDo-03 bacteria exist extracellularly.

### 2.11. Gene Expression Studies in Primmorphs

**Figure 12:**
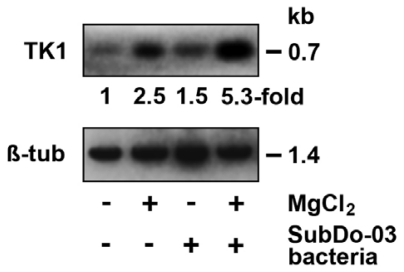
Effect of a co-incubation of BAC-SubDo-03 on the gene expression of the *cytoplasmic thymidine kinase-1* (TK1) gene in sponge primmorphs. Dissociated cells from *S. domuncula* were allowed to form primmorphs in the absence (−) or presence of 50 μM MnCl_2_ (+MnCl_2_) as described under “Experimental Section “. After one day in culture, one set of primmorphs remained non-inoculated with BAC-SubDo-03 (−), or was incubated for three days with those Mn-oxidizing bacteria (+ SubDo-03 bacteria). Then RNA was extracted from the aggregates, and subjected to Northern blot analysis to assess/estimate the level of expression of the gene *cytoplasmic thymidine kinase-1* (a cell-cycle regulated gene) and of *β-tubulin* (house-keeping gene). The expression level of the *thymidine kinase-1* was normalized with the one of *β-tubulin*, as described under “Experimental Section”.

Primmorphs were incubated in the absence or presence of 50 μM MnCl_2_. In parallel assays those 3D-cell aggregates remained either non-inoculated with Mn-oxidizing bacteria, or were inoculated with BAC-SubDo-03, as described under “Experimental Section”. After an incubation period of three days the primmorphs were harvested and RNA was extracted. The transcripts were size-separated and probed with the cDNA encoding the cell-cycle regulated *cytoplasmic thymidine kinase-1* [27,28]. This *thymidine kinase-1* gene is transcriptionally regulated and its expression is limited to the S phase of the cell cycle. In parallel, the expression level of the house-keeping gene *β-tubulin* [29] was assessed. In turn, the level of expression of the *thymidine kinase-1* gene was normalized with the expression of *β-tubulin*. The normalized gene expression of *thymidine kinase-1* was set to 1-fold in primmorphs, incubated in the absence of MnCl_2_ (Figure 12). Addition of 50 μM MnCl_2_ to the cultures resulted in an increase of the expression to 2.5. If the 3D aggregates were inoculated with BAC-SubDo-03 and cultured in the absence of MnCl_2_ the expression of the *thymidine kinase-1* gene only slightly increased to 1.5-fold. However, a simultaneous addition of 50 μM MnCl_2_ to primmorphs co-incubated with BAC-SubDo-03 resulted in a marked increase of *thymidine kinase-1* expression to 5.3-fold. From these findings we conclude that the co-existence of sponge cells with Mn-oxidizing bacteria BAC-SubDo-03 and MnCl_2_ resulted in a pronounced stimulation of growth of the cells in the primmorphs, an argumentation that was also supported by the obvious size increase of primmorphs that had been inoculated with BAC-SubDo-03 and cultures in MnCl_2_-enriched medium (Figure 11).

## 3. Discussion

In the present study a distinct Mn(II)-oxidizing bacterium has been isolated from the sponge *S. domuncula*. It is known that in the collection area of *S. domuncula,* some regions are characterized by elevated levels of Mn ranging from 200 to 800 mg/kg dry weight and a mean of 370 mg/kg dry weight in their coastal sediments [30]. Even though these levels can be exceeded by a factor of 10 to 15 in some regions, for example, in the Baltic Sea or in the Chesapeake Bay [31], the measured levels of Mn could be toxic to an animal. The toxicity level of Mn ranges from 0.01 mg Mn/l for hatching yellow crab embryos (*Cancer anthonyi*) to >500 mg/L for adult Indian Catfish (*Heteropneustes fossilis*). For sponges the toxicity is unknown. At lower concentrations, Mn is an essential nutrient for microorganisms, plants, and animals [32] and known to activate enzymes in plants [33], or in animals [34]. Hence the organisms need a control system that balances and maintains homeostasis in respect to ionic and colloidal- and/or complexed Mn. The most efficient way to keep that balance from exceeding tolerated Mn concentrations is to deposit Mn in a solid form and to release it to the soluble phase as needed under Mn deprivation. A related homeostatic control mechanism exists in sponges and other Metazoa for iron, with the iron/ferritin (iron-storage protein) system [35]. Usually, the concentrations of dissolved Mn (Mn(II)) in seawater are very low and range from 0.08 nM to 10 nM. Similarly, the concentration of dissolved iron in seawater is low (<10 nM). Most of the iron is associated with particles [36]. Close to the sea surface free iron rapidly precipitates/oxidizes to ferric hydroxides [Fe-(OH)_3_] [37]. In contrast to seawater, the concentrations that are measured in blood of vertebrates are much higher, for manganese approximately 0.5 μM [38] and for iron in the range of 10 μM [39].

No Mn-storage system has been described for Mn in animals in general and sponges in particular. The present study suggests that Mn-precipitating bacteria contribute to Mn storage in sponges. The suspected Mn-depositing bacteria were isolated from *S. domuncula* under different culture conditions. Mn(II)-oxidizing *Bacillus* strains were isolated from *S. domuncula*. Among them BAC-SubDo-03 was studied in greater detail. By applying the PCR technique and targeting the rDNA sequence it was established that BAC-SubDo-03 shared highest sequence similarity to Mn-oxidizing bacteria, described by the Tebo group [11–13]. Their spores especially exhibited pronounced Mn-precipitating activity [12]. Spore-forming bacteria are known to possess on their cell walls ectoenzymes [40] that have been implicated in the oxidation of Mn(II) to Mn(IV), with the consequence that Mn precipitates [3,41]. It is remarkable that the surface of some bacteria displays nanostructures and charged ions/molecules that act as bio-seeds for Mn deposition [3,5]. In studying the *S. domuncula*-associated bacteria (BAC-SubDo-03) for their capacity to oxidize Mn(II) and in turn precipitate the oxidized manganese, the existence of an MCO on the cell wall of BAC-SubDo-3 was discovered. Using an *in situ* gel analysis system, a protein species with a size of 110 kDa, and deriving from the envelope of the microorganisms, was identified from BAC-SubDo-03, which retained its oxidizing capacity.

After having identified the Mn-oxidizing capability of the BAC-SubDo-03 bacteria, the effect of Mn(II) on the bacterial growth rate was determined. The studies followed earlier indications that Mn has a positive effect on viability, growth and metabolic activity of bacteria [2]. Similarly well known, is the inducing effect of Mn on spore formation [42]. The growth rate of the *S. domuncula* BAC-SubDo-3 was found to be significantly upregulated in the presence of Mn. When 100 μM MnCl_2_ was added to the culture a significant increase in cell density was seen 10 hours after addition of the ions. It is interesting that, in parallel with its growth stimulating effect, Mn stimulated increased sporulation of BAC-SubDo-3. Addition of Mn(II) to BAC-SubDo-3 shortened its lag phase by 50% and accelerated initiation of sporulation after 10 hours. Similarly strong was the effect of manganous ions on the maximum concentration of spores formed by the culture. Whereas in the absence of Mn(II) only about 20% spores were counted after 50 hours of incubation, in the presence of added Mn(II), 50% spores were counted. On the basis of these findings a stronger Mn(II) oxidation by the bacteria resulting in a larger deposition of manganese oxide on their cell surface and in the culture medium was expected. These findings substantiate the designation of BAC-SubDo-3 as a Mn-precipitating organism.

Bacterial *MCO* were recently cloned [12,13]. Using degenerated primers constructed on the basis of those sequences and directed against the MCO domains of the *mnxG* gene, we identified and cloned the corresponding gene from strain BAC-SubDo-03 and labeled it *mnxG-SubDo-03*. Sequence comparisons revealed that the polypeptide MnxG-SubDo-03, deduced from *mnxG-SubDo-03*, and obtained from the sponge-associated BAC-SubDo-03 shared highest sequence similarity to the Mn-oxidizing bacteria described by the Tebo group [12].

In addition to the cell-wall associated Mn-oxidizing enzyme of bacteria [2], a second system has been identified in bacteria that facilitates Mn precipitation: The S-layers that surround many Archaea and Bacteria [43]. In this system exposed anionic residues are scavenging metal ions [44] and in turn induce biomineralization [45]. Studies on the morphology of the BAC-SubDo-3, both of the vegetative cells and the spores are now in progress stimulated by our finding that the 110 kDa protein of the envelope of the spores is involved in Mn oxidation. A protein with the same size had been identified as a core protein of S-layers [44].

In the present work, strong emphasis was put on the question whether Mn induces expression of the *mnxGpSubDo-03* gene. An answer to this problem would clarify if the BAC-SubDo-03 bacteria could sense extracellularly present Mn(II) ions, and react to that level by an altered *MCO* gene expression. The results were clear-cut. In the absence of Mn in the medium the expression of *MCO*, if existing at all, was not measurable under the conditions used here. However, a short pulse of Mn(II) for 30 minutes resulted in a strong burst of *MCO* transcripts in BAC-SubDo-03. Nevertheless, this high level of *mnxG-SubDo-03* transcripts was not maintained, since already after an exposure for 60 minutes their level dropped considerably and no transcripts were identified after a longer Mn exposure period. This finding supports the well recognized fact that the induction of a bacterial/archaebacterial gene requires only a few minutes of presence of the respective inducer, with the half-life of the resultant transcript being less than 30 minutes [46–49]. Based on this finding, we propose that expression of the *MCO* gene is under a tight environmental control, a process which might allow tuned responses of the bacteria to changing extracellular Mn concentrations.

As was initially proposed [48] and soon accepted by others [50], bacteria live in a symbiotic/commensal relationship with sponges. Most of the sponge species living in the Mediterranean Sea contain, if they are kept in an aquarium for more than two weeks [51], only a few different bacterial strains which are not abundant and, with regard to *S. domuncula*, located in specific cells, the bacteriocytes [26]. This is in contrast to a few species like *Aplysina aerophoba*, which contain up to 50% of the sponge body mass as microorganisms [52]. This finding suggests that the bacteria isolated from *S. domuncula* display a crucial role in the physiology/metabolism of the sponge, perhaps supporting our assumption that the Mn-precipitating bacteria are responsible for reversible manganese storage in *S. domuncula*. This assumption has been experimentally supported by *in vitro* experiments using the primmorph cell system. If those cultures were incubated with the BAC-SubDo-03 bacteria in the absence of Mn(II) no considerable change of the size of the aggregates and alteration of the gene expression for the cell-cycle regulated *cytoplasmic thymidine kinase-1* [27,28] could be determined. However, if those primmorphs, inoculated with BAC-SubDo-03, were grown in medium supplemented with Mn(II), a strong increase in the size of the aggregates is seen, which is paralleled by an upregulation of the expression of the *thymidine kinase-1*. In addition, it could also be demonstrated that the bacteria BAC-SubDo-03 remained inside the primmorphs after an incubation period of three days and were not eliminated from them [53].

**Figure 13:**
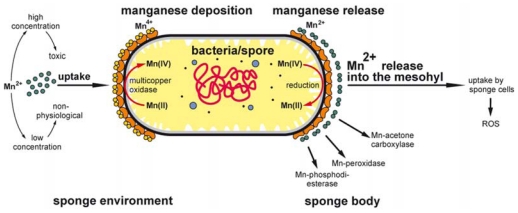
Schematic representation of the proposed role of the BAC-SubDo-03 *Bacillus* strain, associated with *S. domuncula*, as an agent in Mn storage. It is assumed that under high Mn concentrations the bacteria (vegetative cells, sporangia, and spores.) take up Mn(II) from the environment through the multicopper oxidase (MCO) and deposit the ions as insoluble Mn(IV) on their cell wall. If Mn exists in the environment only at low concentrations the MCO allows the enrichment of the element to physiological levels. Intracellularly, in the sponge body, Mn is solubilized by reduction from Mn(IV) to Mn(II) released from the cell wall and becomes available as co-factor in a series of essential enzymes involved in detoxification of reactive oxygen species (ROS), or in conversion of acetone to acetoacetate (e.g., Mn-acetone carboxylase) or lipid metabolism (Mn-phosphodiesterase).

In turn, the bacteria appear to be essential for the maintenance of the physiological Mn concentrations in the sponge. Since only minute levels of Mn exist usually in the surrounding seawater a substantial accumulation of Mn on the surface of the bacteria is proposed. It can be postulated that, if required, the release of bacterial-precipitated Mn(IV) suffices the physiological need for Mn. According to the reaction mechanisms described recently for the MCO from *Escherichia coli*, and based on the experimental proven four-electron reduction of dioxygen by the MCO [54], a reduction and, in turn, a dissolution of Mn(IV) to Mn(II) may be postulated (Figure 13). Furthermore, we postulate that the presence of BAC-SubDo-3 bacteria is required as a protection against higher, toxic concentrations of Mn; after oxidation of Mn(II) to Mn(IV) the ion becomes insoluble [20].

## 4. Experimental Section

### 4.1. Materials

Reagents were purchased as follows: Sterile seawater, Mn Agar (cat no. M2053; containing beef extract (1 g/L), yeast extract (75 mg/L), Mn-carbonate (2 g/L (10 mM)), ferrous ammonium sulfate (150 mg/L), Na-citrate (0.15 g/L) and leucoberbelin blue from Sigma, Taufkirchen (Germany); malachite green oxalate, safranin O, bacto-peptone, yeast extract, Mn(II)-chloride tetrahydrate, Triton X-100 and 2-(*N*-morpholino)-ethanesulfonic acid [MES] came from Carl-Roth, Karlsruhe (Germany).

### 4.2. Isolation and Cultivation of Manganese Oxidizing Bacteria from *S. domuncula*

Specimens of the marine sponge *Suberites domuncula* (Porifera, Demospongiae, Hadromerida) were collected in the Northern Adriatic Sea near Rovinj (Croatia), and then kept in aquaria in Mainz (Germany) at 17 °C. For the isolation of the Mn-oxidizing bacteria only specimens that lived at least for 6 months under these conditions were used.

Tissue samples (200 mg) were taken and minced with seawater (1:1; wt/vol). Serial dilutions of the resulting suspension (10^−1^ to 10^−5^) were prepared and aliquots of 0.1 mL were placed on Mn Agar plates (100 μM of MnCO_3_) [55]. Plates were incubated at 28 °C for 48 up to 72 hrs during which the bacteria were allowed to oxidize Mn(II) ions. Brown colonies, indicative for Mn(II)-oxidizing bacteria, were picked from the agar plates and transferred to a modified K-medium (pH 6.5; 1 l consisting of 75% natural seawater, 2 g bacto-peptone, 0.5 g yeast extract, and 100 μM MnCl_2_) as described [21]. In parallel assays, the microorganisms were cultivated on agar plates or in modified K medium lacking Mn. Sub-cultivation was performed by 1:10 dilution with fresh medium. Among the different

Mn-oxidizing bacterial strains (termed BAC-SubDo), strain BAC-SubDo-03 was selected because of its simple growth characteristics. In one series of experiments BAC-SubDo-03 was incubated in the presence of different concentrations of MnCl_2_ (0 to 200 μM) in the absence or presence of 1.3 to 5 μM cupric chloride (CuCl_2_). The cell concentration was kept constant within a set of experiments.

To check if the isolate BAC-SubDo-03 existed also in the seawater of the aquarium, 3 mL water samples were taken for PCR analysis and spun down at 11,000×g for 10 min at 4 °C. The pellets were resuspended in polymerase chain reaction (PCR) buffer and analyzed for the presence of the *mnxG-SubDo-03* gene by PCR; see below. In a further series of experiments DNA was isolated from the seawater, following the CTAB (cetyltrimethylammonium bromide)-NaCl extraction procedure, described by us [24,56].

### 4.3. Isolation and Purification of Spores from the Bacterial Cultures

Cultures of BAC-SubDo-03 were set up in modified K medium (in the presence of 100 μM MnCl_2_) and incubated at 28 °C. Under those conditions BAC-SubDo-03 started to form spores (endospores) after 10 hrs, as described also for other bacteria [21]. In parallel, BAC-SubDo-03 cultures were grown in modified K medium lacking MnCl_2_. In order to quantify the fraction of spores in the culture, samples were taken and stained applying a described procedure [57]. Visualization of the spores was achieved by staining with 7.6% malachite green (in distilled H_2_O) and after counterstaining with 0.5% safranin. Microscopic analysis was performed with an Olympus AHBT3 light microscope.

In addition, the morphology of the spore-forming bacteria was monitored, by scanning electron microscopy (SEM) analyses. Samples were fixed in 0.1% glutaraldehyde (pH 7.9; in 75% seawater and buffered with 50 mM HEPES) overnight. Then, the samples were mounted onto aluminum stubs (SEM-Stubs G031Z; Plano, Wetzlar; Germany) and subjected to SEM with a Gemini Leo 1530 high resolution field emission scanning electron microscope (Oberkochen; Germany).

### 4.4. Detection of Mn(II)-Oxidizing Activity in Spores

Two assay systems were applied to verify that the BAC-SubDo-03 strain exhibits Mn(II)-oxidizing activity: (*i*) the detection of the Mn-oxidizing activity in the cell wall of the microorganisms by the *in situ* “in-gel oxidation assay”, and (*ii*) the detection of the conversion reaction of Mn(II) to Mn(IV) by the bacteria in the growth medium, applying an optical test.

The Mn-oxidizing activity of the outermost spore layer(s) was determined by the *in situ* “in-gel oxidation assay” [22,58]. The bacteria were pelleted by centrifugation (10,000×g; 4 °C), washed with deionized water, and suspended in 10 mM Tris buffer (pH 7.5). Then, the cells were treated with lysozyme (50 μg/mL; 30 min at 37 °C) to obtain spores. Subsequently, the samples were washed (five times) with deionized water (supplemented with 10 mM EDTA and 5% phenylmethane sulphonyl fluoride [pH 7.5]) and the spores were collected by centrifugation (10,000×g; 4 °C) and subsequently homogenized at 6,500 rpm in a Precellys 24 homogenizer (PeqLab Biotechnologie, Erlangen; Germany). The broken spores were subjected to sodium dodecyl sulphate polyacrylamide gel electrophoresis (SDS-PAGE) using 10% gels. After size separation the gels were stained for total protein with Coomassie brilliant blue. To assess Mn(II) oxidation activity a described procedure was used. At first, the gels were incubated for 30 min in a 10 mM Tris buffer (pH 7.5; 0.5% Triton X-100, 10% [v/v] glycerol) to remove the NaDodSO_4_ and then the gels were incubated in 10 mM HEPES buffer (pH 7.6; 200 μM MnCl_2_). The process of Mn(II) oxidation could be followed visually by the formation of brown Mn oxide bands on the gels. Those bands usually appeared after 2 hrs of incubation. Previous data indicated/demonstrated that the Mn(II)-oxidizing activities, mediated by spore surface proteins, is due to the copper (Cu)-dependent MCO [12,58]. Therefore, we used the Cu-ion chelator o-phenanthroline to check if the Mn-oxidation displayed by BAC-SubDo-03 spores can be attributed to a MCO as well. Consequently, incubation of the gels was performed with 50 μM of *o*-phenanthroline (in 10 mM HEPES buffer at pH 7.6) for 15 min prior to the addition of 200 μM MnCl_2_. Under those conditions the color reaction of the band in the gel was totally suppressed.

The Mn(II)-oxidizing reaction proceeding in the medium was followed by an optical assay that is based on leucoberbelin blue that binds to Mn(III) and Mn(IV) [21,59]. The bacterial cultures grown in K-medium (supplemented with 50 μM to 200 μM MnCl_2_) for 6 to 24 hrs were centrifuged and the sediment was used for the quantitation of Mn(IV). Aliquots of 0.05 to 0.25 mL were taken from the cultures and added to 0.5 mL of a 0.04% (w/v) leucoberbelin blue reagent. In the presence of Mn(III) or Mn(IV) leucoberbelin blue is oxidized with a concomitant appearance of a blue color [59]. This shift was monitored optically at 620 nm. K-permanganate was used to establish a calibration curve.

### 4.5. Determination of Binding/Oxidation Capacity of BAC-SubDo-03 Strain for Manganese

BAC-SubDo-03 were cultivated in modified K medium (in the presence of 100 μM MnCl_2_) at 28 °C for 24 hrs. Then the microorganisms were transferred to new modified K-medium (containing 100 μM MnCl_2_), supplemented with radiolabeled manganese (^54^Mn^2+^; Amersham, Arlington, Heights, Ill.; U.S.; specific activity of 2.66 × 10^6^ Bq/μg). To 1 mL of culture 74 × 10^4^ Bq of ^54^Mn^2+^ were added and cultivation was carried on at 28 °C for 50 min. Then aliquots of 100 μL were taken, filtered through filters (Whatman GF/C glass filters), transferred to counting vials that were filled with 4.5 mL scintillation cocktail, and then radioactivity was determined as described [23,60]. By using a standard the counting efficiency was determined. The values are given in nmoles of Mn bound/oxidized/mg of protein.

### 4.6. Cloning and Sequencing of Bacterial 16S rDNA by PCR

Single bacterial colonies were picked and the DNA was isolated by the “AxyPrep multisource Genomic DNA Miniprep Kit” (Axygen Biosciences, Union City, CA; U.S.), as described [61].

For polymerase chain reaction (PCR) amplification of 16S rDNA fragments we applied widely used primers [62]; forward primer 27f (5′-GAGTTTGATCCTGGCTCAG-3′ together with the reverse primer 1100r (5′-GGGTTGCGCTCGTTGC-3′). The PCR product spanned the nucleotides [nt] 9–1,113 of the *Escherichia coli* 16S rDNA gene (*E. coli* 16S rDNA sequence; accession number J01859).

The conditions for the PCR were as follows. The 50 μL reaction contained the undiluted PCR buffer (Peq*-*Lab), 2 mM MgCl_2_, 1.5 U of Taq DNA polymerase (Peq*-*Lab), 0.2 mM dNTPs (Roth), 10 pmol of each primer, and 1 to10 ng of template DNA. PCR cocktails were subjected to 35 cycles of amplification in a PCR thermal cycler (iCycler, Bio-Rad). The cycling conditions were as follows: initial denaturation (2 min at 95 °C), followed by 35 cycles each of denaturation (25 sec at 95 °C), annealing (30 sec at 52 °C) and extension (1 min at 72 °C), and a final extension step (7 min at 72 °C). After the cycling 5 μL aliquots were resolved in 1% agarose/TAE [Tris-acetate-EDTA] gel, stained with ethidium bromide and visualized by UV-illumination.

PCR products were purified from the reaction mixture by the “NucleoSpin Extract II” kit (Macherey-Nagel, Düren; Germany) as recommended by the manufacturer. Purified PCR products were cloned into pGEM-T vector (Promega, Madison, WI; U.S.). Plasmid DNA was isolated by “High Pure Plasmid Isolation Kit” (Roche, Mannheim; Germany). Sequencing was performed by Thermo Sequenase DNA polymerase “Fluorescent Labelled Primer Cycle Sequencing” kit with 7-deaza-dGTP (Amersham Pharmacia Biotech, Freiburg; Germany) using primers, labeled with IRD-700 or IRD-800. Sequencing ladders were resolved in SequaGel XR gel (National Diagnostics, Atlanta, GA; U.S.) using a Li-Cor Sequencer-4300.

To eliminate possible PCR errors several clones were sequenced and a consensus was compiled. The resulting sequence of the 1,114 bp long 16S rDNA fragment was termed 16S-BAC-SubDo-03.

### 4.7. Cloning and Sequencing of Multicopper Oxidase *mnxG* Gene by PCR

Genomic DNA of the strain BAC-SubDo-03 was isolated as described above. In order to isolate the *mnxG* gene from the sponge-associated microorganism BAC-SubDo-03 degenerated primers that had been designed according to published sequences of *mnxG* genes were used [11–13]. The expected PCR product of approximately 700 bp spans three out of the four/six copper-binding domains that are found in the *mnxG* cluster of *Bacillus* sp. strain SG-1 and some other Mn-oxidizing bacteria [10,11,13]. The forward primer was targeted to domain D (“HCHL-F” 5′-CAYTGYCAYYTRTAYCCNCA-3′ nt pos. 4313 to 4332 in strain SG-1 [accession number U31081.1]) and the reverse primer (“HQQH-R” 5′-CCAAATAYWCCATGYTGYTGRTG-3′ nt pos. 5079 to 5057 in SG-1) was targeted to domain B. The PCR mixture was composed as described above but containing 100 pmols of each of the *mnxG* primers. The PCR cycling conditions were as follows: initial denaturation for 2 min at 95 °C, and then 35 cycles each of denaturation (25 sec at 95 °C), annealing (30 sec at 47 °C) and extension (1 min at 70 °C). The final extension step was performed for 7 min at 72 °C. The completed PCR reactions were resolved in 1% agarose/TAE gel, stained in ethidium bromide and visualized by UV-illumination. PCR products of the expected size (approximately 700 bp) were cut out from the gel, cloned and sequenced as described above. The consensus derived from several sequenced clones of the mnxG gene from the S. domuncula-associated *Bacillus* strain BAC-SubDo-03 is 708 bp long and has been termed *mnxG-SubDo-03.*

### 4.8. Expression Studies of *mnxG-SubDo-03* Gene by RT-PCR

The expression of the *mnxG-SubDo-03* gene was semi-quantitatively assessed by RT-PCR (reverse transcription-PCR), selecting a segment of the *mnxG* gene as a target. Bacterial cultures in K-medium (without MnCl_2_) were inoculated from fresh colonies and cultivated at 28 °C with shaking. At the indicated times after inoculation cultures were supplemented with 100 μM MnCl_2_. Aliquots were taken and the cell density in each assay was adjusted to the same OD (2.0 absorbance at 660 nm). Starting with 5 mL of culture, total bacterial RNA was isolated by “AxyPrep Multisource Total RNA Kit” (Axygen). Satisfactory yields of RNA were obtained from BAC-SubDo-03 by homogenization of the cells in a “Precellys-24” bead grinder (PeqLab). For the homogenization “Axygen Cell Lysis Buffer R-I” was used together with 1 mm glass beads. Grinding was performed 3 times (5 sec each at 6,000 rpm). To remove DNA, isolated RNA was treated with DNAse (Ambion Inc) at 37 °C for 30 min. Subsequently, DNAse was removed by DNAse inactivation reagent (“DNA-free” kit, Ambion). cDNA was synthesized by M-MLV Reverse Transcriptase [RT] (RNAse H Minus) primed by random decamers in 40 μL final volume, as recommended by the manufacturer (Promega). Each reaction contained 10 μg of RNA, 0.5 mM dNTPs and 400 U of the RT enzyme. The reaction mixtures were incubated at 42 °C for 1 hr; the RT was inactivated by incubation at 65 °C for 15 min. The following primer pair was used for the amplification of the *mnxG* gene: gmnx-F 5′-ATGTGGGGTATCAATCGGATC-3′ (nt 34 to 54 in *mnxG-SubDo-03*; accession number FN565386) and qmnx-R 5′-GTAGGACTATGGTGGACAAGG-3′ (nt 152 to 133). One μg of transcribed RNA was added per reaction. PCR cycling conditions were as following: 95 °C-20 sec; 56 °C-30 sec; 70 °C-45 sec for 40 cycles. The size of the PCR product was 119 bp. Aliquots of 5 μL were loaded onto high resolution gels (4 cm long gels, prepared from 3% MS500 agarose (PeqLab) in 1 × TBE [Tris base-boric acid-EDTA; pH 8.0]. As a control the PCR experiments were performed with samples that had been obtained form RNA preparations that had not been subjected to RT reaction.

### 4.9. Data Analysis

The sequences were analyzed with computer programs Blast (2005; http://www.ncbi.nlm.nih.gov/blast/blast.cgi) and FASTA (2005; http://www.ebi.ac.uk/fasta33/). Multiple alignments were performed as outlined in detail [63] and phylogenetic trees were constructed based on sequence alignment by neighbor-joining. The distance matrices were calculated using the Dayhoff/PAM matrix model as described [64]. The degree of support for internal branches was further assessed by bootstrapping. The graphic presentations were prepared with GeneDoc [65].

### 4.10. Primmorphs and Co-Incubation with BAC-SubDo-03

Primmorphs were obtained from single cells that had been obtained by dissociation from sponge tissue, as described [19]. The cells were transferred to natural seawater supplemented with 0.2% RPMI1640 medium. Where indicated the medium contained 50 μM MnCl_2_. After one day the cultures were split into 1 mL assays and transferred into 6-well plates (Nunc, Langenselbold; Germany) and incubation was continued either in Mn-free or in 50 μM MnCl_2_ containing medium. In parallel, primmorph samples were co-incubated with the bacteria BAC-SubDo-03. Aliquots of 50 μL from an overnight culture of BAC-SubDo-03 grown in modified K-medium in the absence of MnCl_2_ (OD600nm of 1.5) were added to the primmorphs. Three days later the 3D-cell aggregates, the primmorphs, reached sizes of 2–6 mm and were used for histological and gene expression analyses.

### 4.11. Histological Analysis

Primmorphs were fixed in paraformaldehyde, embedded in Technovit 8100 and sectioned, essentially as described [66]. The 5 μm thick slices were stained with hematoxylin/eosin [67] and inspected with an Olympus AHBT3 microscope.

### 4.12. RNA Extraction and Northern Blotting

RNA was extracted from liquid-nitrogen pulverized primmorphs with TRIzol Reagent (GibcoBRL Grand Island, NY; U.S.) as recommended by the manufacturer. An amount of 3 μg of total RNA was electrophoresed through formaldehyde/agarose gel and blotted onto Hybond N+ membrane as described before [68]. Hybridization experiments were performed with the following probes: the *S. domuncula thymidine kinase* cDNA (nt_51–162_ a nucleotide segment localized within the coding region of the deduced protein; accession number CAP19646 [27]) and the *S. domuncula β-tubulin* cDNA fragment (nt_83–423_; CAD79598 [69]). The probes were labeled with DIG-11-dUTP by the DIG DNA labeling kit (Roche Diagnostics, Mannheim; Germany). After washing, DIG-labeled nucleic acid was detected with anti-DIG Fab fragments (conjugated to alkaline phosphatase) and visualized by the chemiluminescence technique using CDP-Star. Hybridization was performed under high stringency as described [70]. For the semiquantitative analysis of the expression level, the bands on the film were scanned with the GS-525 Molecular Imager (Bio-Rad, Hercules, CA; U.S.). The signals on the blots were quantified and the intensities reflecting the *thymidine kinase* transcripts were correlated with those for *β-tubulin*. The expression fractions (ratios between the intensities of the signals for *thymidine kinase* and those of the corresponding signals for *β-tubulin*) were calculated. The expression fraction measured for primmorphs which had been incubated without MnCl_2_ and had not inoculated with BAC-SubDo-03 was set to 1-fold. Five parallel experiments were performed. The values did not differ by more than 20%.

### 4.13. Analytical Method

For the quantification of protein the Bradford method ([71]; Roti-Quant solution - Roth) was used.

### 4.14. Statistical Analysis

The results were statistically evaluated using the paired Student’s *t*-test [72].
